# Adverse impact of lockdown during COVID-19 pandemic on micro-small and medium enterprises (Indian handicraft sector): A study on highlighted exit strategies and important determinants

**DOI:** 10.1186/s43093-022-00166-0

**Published:** 2022-11-08

**Authors:** Uma Shankar Yadav, Ravindra Tripathi, Mano Ashish Tripathi

**Affiliations:** Department of Humanities and Social Sciences, Motilal Nehru National Institute of Technology Allahabad, Uttar Pradesh, Prayagraj, India

**Keywords:** COVID-19 pandemic, Cavite, Handicraft sector, Descriptive research, Exit strategies, Small enterprises, Determinant, Adverse impact

## Abstract

The COVID-19 pandemic had a significant impact on the global economy. For small- and medium-sized businesses, particularly in the handicraft sectors, it was exceptionally difficult to maintain operations during a global crisis. Without crisis management plans in place, business owners run the risk of having their operations abruptly terminated. Therefore, the researchers sought to comprehend and study the factors that led to the shutdown and departure strategies of microenterprises (in the handicraft sector) during the pandemic. To conduct this study, a descriptive research design was used, and registered Indian micro-businesses in the Cavite region were specifically chosen. According to the study's findings, the majority of microenterprises were sole proprietorships with a capitalization of less than INR 600,000.00 (Asian Development Bank in The COVID-19 impact on Philippine businesses: key findings from the enterprise survey*.* http://dx.doi.org/10.22617/SPR200214-2, 2020) and had been in business for three to six years. As a result, the respondents' most typical issues are Retooling and training of pandemic-sensitive enterprise strategies for micro-entrepreneurs, particularly in the handicraft sector, as well as research into the negative effects of lockdown on MSME, which are all examples of crisis-sensitive interventions among small industries (handicraft sector) entrepreneurs.

## Introduction

The COVID-19 crisis is viewed as a sudden threat that impacts not only a whole organization but also an entire economic sector. As pointed out in the IEA and UNEP reports, the COVID-19 pandemic has profoundly affected and changed global and regional economic and environmental issues. Therefore, this section should not ignore the impact of the pandemic on environmental and economic issues Wang and Su [1]. To survive in the new normal during the pandemic breakout, recovery is essential. Large businesses in industrialized countries have been studied for their business continuity plans. Microbusinesses have, however, received relatively little attention, especially in poor countries. Small organizations can experience unexpected operational disruption as a result of the absence of efficient crisis management techniques [2]. Due to the COVID-19 epidemic, businesses are more susceptible to crises. This affected both the corporate sector and the lives of Indians. Local government organizations in India responded right away before the outbreak started. Due to the crisis that resulted in business closures and losses, quarantine measures were put in place to guarantee both the community's safety and the economy [3]. In the National Capital Region, a survey by the Asian Development Bank [4] found that two-thirds of enterprises temporarily closed, with most others (29%) decreasing operations. Only 4% of the businesses continued to function at full capacity, while the majority (78%) still operated at half capacity or less. Additionally, the application of customary practices. Research on the effects of developing crises, like a pandemic outbreak, on micro-enterprises is still lacking. Microbusinesses are thought to be more susceptible to restrictions. Thus, this study focused on identifying and understanding the determinants of microenterprises shutdown and exit strategies during the COVID-19 pandemic. In reality, a few other nations, like India, see an increase in their usage of renewable energy as a result of the economic slowdown in the EU. However, the EU economic recession's knock-on effects result in a decline in manufacturing due to the pandemic COVID-19 as stated by Rongrong et al. [5]. Moreover, this study served as a guide for microenterprises' business survival strategies to keep their operations running despite the new normal. The relevant information for handicraft entrepreneurs would assist them in managing their businesses in an outbreak of COVID-19. Furthermore, this can serve as a baseline for government agencies to intervene in a more pandemic-sensitive manner.

### Bad effects of pandemic COVID -19 on micro-small and medium enterprises especially in the handicraft sector

Even all sectors were affected by pandemic 19 when a nationwide lockdown was declared by governments. But data has shown that the handicraft sector as a part of the small industry was much affected by shutdown and labor, and artisans suffered much more. Their hands were empty, and there was no export of their handmade products. Labor section also faced problems because no owner gave the work to them, even rural people and especially women were engaged in making handmade mask during these days and helped the governments and they upgraded their skill and in the local term, they enhanced their Hunnar(talent) [6]. Digital technology also affected this sector because most of the work is done in traditional patterns and workers and artisans are less aware of digital knowledge. But, on the other hand, this sector gave local jobs and earning opportunities to the people.

## Literature review

The following literature supports the current study, like the study done by [7] described the important steps like as digital technology, craft museum, and online training centres, that are useful for the development of Handicraft sector in India. They explained the import of handmade carpets and Shazar stones and he also discussed how this industry was affected by lockdown period in pandemic-19 in last 2 years. Tripathi et al. [8] focused on Gig Workers, labor productivity and textile sector of India and they also discussed in their paper that most of the labors are women in the textile industry. A study conducted by Shinozaki and Rao [3] for 690 handicraft industries (Small business) enterprises found a drastic fall in the growth rate (this was due to pandemic COVID-19 of net sales by (−)66.7% in the first quarter of the financial year 2020–2021. Kumar et al. [9] discussed a visionary concept of the global handicraft index and role of the role of handicraft artisan and strategies for the development of the Khadi and Handloom, Handicraft, Village Industries, Bamboo-Based Industries, Sericulture, and Lock, etc., are traditional small-scale industries [10]. A wide range of products ranging from relatively simple items to sophisticated products such as television sets, electronic control systems, mixer grinders, and various engineering products are produced by modern small-scale industries, particularly as ancillary to large industries and meanwhile [5] observed that COVID-19 has affected the energy sector of China and India so during the time these countries turned toward renewable energy. Traditional small businesses are highly labor-intensive, whereas modern small-scale units use highly advanced machinery and equipment. The situation worsened further when the government announced the extended nationwide lockdown amidst the COVID-19 crisis [11]. They suggested that there is an enormous gender disparity in employment; that is women are very few in comparison to men workers, so we should have to resolve this disparity in MSME sector. Yadav et al. [7] published their research paper "Study of Handicraft Marketing Strategies of Artisans in Uttar Pradesh and its Implications" as we have discussed the performance of the handicraft sector and the role of women in the handicraft sector or home-based industry. The following literature supports the objective of the article. Parilla [12] has done his study and focused on the role of the handicraft sector. He observed that Indian artisans can create a variety of designs and introduced four P as promotion, place, Price, and in the last Production. Goswami and Goswami [13] focused that handmade products run with help of SHGs and NGOs involved in this sector with the support of the corporate sector and some grants and subsidy along with funds of related ministries in the country as well as the state of Uttar Pradesh and different NGOs in the handicraft sector. Parilla [12] discussed the Role of Self-help groups (SHGs) and some private organizations called NGO for marketing and production of craft in Philippine and suggested that these NGOs and Grupus have enhanced the marketing and production capacity of craft products. According to [1] COVID-19 has largely created an adverse impact on the economies and environment; obviously, MSMEs were also affected during this time.

Andal et al. [14] has discussed the diverse effect of the pandemic on the MSE industry in the last 1 year. He suggested that financial support to the handicraft industry should be given by the government. Tambunan [15] focused on the role of a contributor to the Indian craft business in his article. His empirical analysis points out the role performed by the handicraft industries and he further explained to explore many threats and risks for small and female-based industries. This researcher used, and experimental, descriptive, and analytical methodologies. Khan and Amir [16] and Mohi-us-din [17] in his research paper have analyzed the Positive impact of handmade products like craft, textile, handloom, woodcraft, pottery, terracotta, Jamdani, and embroidery education in vocational training through handicrafts based on traditions and local customs. An advanced and most effective technique may have a big impact on the learning and teaching method of handicrafts, as well as their existence, and identity preservation of their handmade skill characters [18].

According to [19] handicraft experts observed that academicians, researchers, and students can learn the value and potential of craft in terms of marketing economy, supply methods and utilize their knowledge, aptitude, and skill to increase the income of workers, artisan involved in the handicraft industries. The findings show that there is a large gap in the worth and quality of craft for the learners in both the control groups and experimental.

But [20, 21] published about the performance of women in ODOP of Uttar Pradesh and they gave an initial approach to the developing global handicraft index for small businesses. A new concept for the development of the handicraft industry in the world and to enhance the positive completion in a new era there is a need for a global handicraft index [18]. We know that women are involved in the handicrafts sector and their performance is increasing day by day even during the pandemic time. So it needs to make strategies for its development in the handicraft industry [6]. In the case of formal and informal knowledge transformation in the handmade carpet industry, analyzed the good criteria for the transformation of institutions [22]. How to develop business strategies for upgrading the handicraft artisan’s skills there is a need for special strategies [7]. Sarkar [23] described important small industries in Azerbaijan and different handicraft industries and how to develop special strategies in the handicraft sector. Some famous Shazar stone industries in Uttar Pradesh are also in the decline phase and we need to improve this Shazar stone sector. Tajudin et al. [24] discussed the digital transformation and innovation of the handmade carpet industry during a pandemic time.

## Objectives of the study

The study generally aimed

- To understand and analyze the determinants of handicraft enterprises’ lockdown and exit strategies applied during the covid-19 pandemic.
- To identify the problems encountered in managing the handicraft enterprise during the pandemic in terms of marketing aspect; financial aspect; operational aspect; and staffing and leadership.
- To identify the exit strategies employed by the respondents, and proposed possible interventions which can help business continuity during the crisis.

## Materials and methods

### Research design

This study used a descriptive research design to understand the determinants and exit strategies of microenterprises. The business profile and identify problems encountered in managing the microenterprises were also understood and analyzed through a descriptive research approach. OECD [25] explained that the current global environment may result in a considerable drop in the amount of money accessible to developing countries. This raises the possibility of severe development setbacks, making it more vulnerable to future pandemics, climate change, and other global public health issues. As stated by [26] due to the global pandemic, most enterprises closed because of policy mandates or decreased demand shifts, and many of these enterprises are closed permanently due to the sustainability of their expenses and are unable to avoid the shutdown [27].

### Sampling Design

The researchers utilized 30 microenterprises that were duly registered with the Department of Trade and Industry and seized the operations during a pandemic. The samples were purposively selected in the municipalities of the program, Lucknow, Prayagraj, Delhi, and Agra.

## Research instrument

A self-constructed survey questionnaire was administered through the use of the Google Form application due to the limitations and restrictions brought by the pandemic. The validity, reliability, and usability of the research instrument were examined and established by a pool of academicians and experts in the business.

### Statistical treatment

The researchers utilized statistical techniques and formulas to analyze the reasons, causes, and exit strategies of micro-enterprises to come up with conclusions. Descriptive statistics were used in this study; frequency count, and percentage distribution.

### Ethical consideration

During the study, the researchers adhered to ethical research practices. Before distributing the survey questionnaires, the proponents obtained permission from the respondents to participate in the study and kept all participant confidentiality private. Anonymity was also maintained to ensure that no physical, emotional, or social harm was done. A crisis is an unpredictable event that affects the revenue, human life, safety, energy and environment, property, and reputation of an organization that would require extraordinary management skills [12]. Moreover, a crisis is defined as a situation faced by an individual, group, or organization that they are unable to cope with through time [6], survey base, and questionnaire method applied during the data collection from respondents.

### Data gathering procedure

The primary data was collected, analyzed, and interpreted through business owners' responses organized using questionnaires and software. The researchers compiled secondary evidence from online published scholarly articles, journals, and academic references relevant to the strategies of micro-enterprises during the COVID-19 pandemic.

## Results and discussion

In this section, we have discussed and analyzed the data, and factors that affected the MSME s industry during the Pandemic. These findings are on the basis of answers given by respondents. In past studies, no exit strategies were described by earlier researchers during such type of crisis or pandemic. So this discussion will focus on the strategies and determinants that have been focused on.

Table 1 shows the type of business ownership of the respondents. This reveals that 23 or 76.67% of handicraft enterprises are sole proprietors, while seven or 23.33% of the participants are partnerships in handicraft sector. The majority of the micro-enterprises were sole proprietorships.

**Table 1:** The Type of handicraft business ownership

Category	Frequency	Percentage
Sole Proprietorship	23	76.67
Partnership	7	23.33
Total	30	100.00

Sources: Table compiled by the author

Table 2 presents the length of the business operation of the respondents. It shows that 21 or 70.00% of the micro-(handmade)enterprises have been operating for two to five years, while two or 6.67% are 10 years above. This reveals that the majority of the respondents have been operating for two to five years.

**Table 2:** Length of small and handicraft business operation

Category	Frequency	Percentage
Below 1 year	4	13.33
2 to 5 years	21	70.00
6 to 9 years	3	10.00
10 years and above	2	6.67
Total	30	100.00

Sources: Table compiled by author

Table 3 presents the average initial capital of the micro-enterprises. This table depicts that 28 of the respondents or 93.33% have initial capital of below INR 500,000, while one or 3.33% have INR 500,001 to 1,000,000 and INR 2,500,001 to 3,000,000, respectively. The majority of the respondents have an average initial capital of below INR 500,000.

**Table 3:** Average initial capital in handicraft business

Category	F	Percentage
Below INR500,000	28	93.33
INR 500,001 to 1,000,000	1	3.33
INR 2,500,001 to 3,000,000	1	3.33
Total	30	100.00

Sources: Table compiled by author

Table 4 shows the problems encountered by the (handmade)-enterprises in managing their enterprises in the handicraft sector in India during the pandemic in terms of handicraft products marketing aspects. This reveals that 24 or 43.64%, were limited to direct marketing due to government restrictions, followed by inefficient promotional activities or strategies with 14 or 25.45%, while the third-highest responses are lack of marketing plan with 8 or 14.55%. Considerably, most of the problems encountered by the respondents with regards to the marketing aspect are the limitation on direct marketing brought by government restrictions. Due to Enhanced Community Quarantine measures, enterprises faced severe difficulties. It restricted the movement of workers and consumers as well as the business operational hours. Although the handmade enterprises were limited to access to assistance programs, still most MSMEs were found to be adaptable and innovative in their coping techniques, with the most popular being the use of online platforms and the customization or creation of new products [21].

**Table 4:** Problems encountered in terms of the handicraft products marketing aspect

Category	f	%
Improper management of customer relationship	2	3.64
Inefficient promotional activities or strategies	14	25.45
Limited direct marketing due to restrictions of government	24	43.64
Lack of marketing plan	8	14.55
Meeting customer satisfaction	1	1.82
Inadequate and irregular supply of raw materials	6	10.91
Total	55	100.00

Sources: Table compiled by author

Table 5 presents the problems encountered by handicraft business owners in managing the enterprise in terms of financial aspects. The table depicts 22 or 26.19% of the responses were large bills to pay, followed by 14 or 16.67% which is owners’ revenue decreases. The third highest is problems in budgeting with 13 or 15.48% same with funding to grow the business. Most of the respondents encountered problems were large bills to pay. According to the [4], during the outbreak, the number of micro-, small- and medium-sized enterprises (MSMEs) fell by 0.4% in India. A prolonged pandemic made it difficult for MSMEs to raise funds from formal financial institutions and could contribute to more potential losses to the Indian economy [28]. Additionally, the majority of the MSMEs suffered financially because of reduced profitability and consumerist of handicraft products.

**Table 5:** Problems encountered in terms of the financial aspect in handicraft business

Category	F	%
Large bills to pay	22	26.19
Owners’ revenue decreases	14	16.67
Unforeseen business expenses	4	4.76
Problem in budgeting	13	15.48
Too much debt of the business	3	3.57
Finding funds to grow the business	13	15.48
Business expenses are greater than income	8	9.52
Mixing business and personal finances	7	8.33
Total	84	100.00

Sources: Table compiled by the author

Table 6 presents the problems encountered by the business owners in managing the enterprise in terms of operational aspects. This shows that 16 or 25.00% of respondents are starting in operating the business from home and using an online platform; secondly there are difficulties in accessing the workplace, offices, factories, or warehouses with 15 or 23.44%, followed by operating for a lesser time duration with 12 or 18.75%. Most of the problems encountered by the respondents in terms of operational aspects are starting with operating the business from home and using an online platform. The study by Shinozaki and Rao [23, 29], and [3] stated that some enterprises practice contactless transactions and work from home to lessen the rapid increase in cases. Moreover, the outbreak of the pandemic resulted to fear, panic, and confusion in the community, and the working time of the employees is also affected [14]. The global pandemic severely affected microenterprises. To survive, businesses need to remain open, but employees need to work from home, and others need to shut down to cut expenses. Although the government provides incentives and assistance funding, these funds for the handicraft sector may only be available for a short period [16].

**Table 6:** Problems encountered in terms of the operational aspect in small and micro-(handmade) business

Category	f	%
Operating for the lesser time duration	12	18.75
Shortage in raw materials	3	4.69
Production disruption	5	7.81
Difficulties in accessing workplaces, offices, factories, or warehouses	15	23.44
Starting by operating the business from home and using an online platform	16	25.00
Customer’s cancelation of orders	7	10.94
There were difficulties in communicating with the customer	5	7.81
Others	1	1.56
Total	64	100.00

Sources: table compiled by the author

Table 7 shows the problems encountered by business owners in managing the enterprise in terms of staffing and leadership. The table shows that 18 or 24.00% of the responses are the move of employees to work from home, followed by owner’s lack of technical knowledge and used to face-to-face interaction with 15 or 20.00%. The employees who experience mental health problems and fear of losing their job have a frequency of 11 or 14.67%, respectively. Considerably, the business should have a new management strategy that can avoid business failure. Business owners may employ multiple synchronous strategies that can help businesses contentious to operate during the pandemic [26, 30]. The study of [29], indicated that Information Communication Technology is a powerful tool in achieving business survivability that affects social capital building, bridging, and self-efficacy, both directly and indirectly. The entrepreneurial activity can be improved and entrepreneurs can minimize the implications of lock-downs with the help of ICT [31]. Moreover, during a pandemic, micro-entrepreneurs must think outside the box to keep their businesses afloat. Superior human resources are required to combat the COVID-19 pandemic, namely individuals who are skilled in both hard and soft skills. Micro(handicraft)-entrepreneurs must have certain qualities or skills, such as being able to recognize market possibilities, not becoming bored easily, working in a team, and being able to communicate verbally and write reports efficiently [32]. Table 8 shows the determinants of the problems encountered by business owners that led to business failure in terms of marketing aspects. 26 or 45.61% were challenged in re-aligning the goals due to the pandemic, followed by 10 or 17.54% lockdown business experience or knowledge and could not maintain competitiveness. This further reveals that most of the cause of the problems encountered by small entrepreneurs in the marketing of handicraft products is re-aligning the goals of the business during a pandemic [16]. According to the adaptive approach, certain micro-enterprises may need to adapt in the case of a crisis, such as producing a product that is urgent to the market or shifting from traditional to online business. Furthermore, the assisted method argues that micro(handicraft)-entrepreneurs may rely on external help, such as from the government and other supply chain partners, in the event of a crisis [4]. Focusing on customers, networking with other entrepreneurs, and digitizing the business are seen as appropriate and rational tactics for micro-entrepreneurs in this study to survive during the pandemic crisis, even though the pandemic crisis has a severe impact on micro-enterprises [31].

**Table 7:** Problems encountered in terms of staffing and leadership

Category	f	%
Poor management skills and competencies	7	9.33
Move employees to work from home	18	24.00
Owner’s lack of technical knowledge and use to face-to-face interaction	15	20.00
Reduction in leisure/break time of employees	4	5.33
Employees change the number of caring duties	1	1.33
Employees were faced with a dramatic increase in workload and job strain	7	9.33
Employees experience mental health problems because of the crisis	11	14.67
Employee’s fear of losing a job	11	14.67
Others	1	1.33
Total	75	100.00

Sources: Table compiled by author

**Table 8:** Determinants of the problems encountered by the respondents that lead to business failure in terms of the financial aspect

Category	f	%
Customer late payments	5	6.17
The inability of business owners to pay ongoing expenses	22	27.16
Customer delayed debt payment	6	7.41
Business owner’s higher personal spending	8	9.88
Business owner’s underpaying taxes	3	3.70
Not paying bills on time	11	13.58
Business owners failed to pay rent and utilities	19	23.46
Business owners failed to pay salaries	7	8.64
Total	81	100.00

Table 8 shows the determinants of the problems encountered by the micro-enterprises that led to business failure in terms of financial aspects. Most of the responses are the inability of business owners to pay ongoing expenses with 22 or 27.16%, followed by business owners failing to pay rent and utilities with 19 or 23.46%. The third highest response is not paying bills on time with 11 or 13.58%. It reveals that most of the causes of the problems encountered by the business owner were their inability to pay ongoing expenses. This affirms the study of [24] restriction of cash flows and lack of customers and supplies were the challenges of handicraft entrepreneurs during the implementation of quarantine measures; majority city of small and microenterprises were seriously affected because of insufficient cash reserves.

Table 9 showcases the determinants of the problems encountered by micro-enterprises that lead to business failure in terms of operational aspects. The majority are the difficulties in making or developing innovative products with 16 or 26.67, followed by the company having temporarily closed its doors due to new protocols and guidelines with 13 or 21.67%. The third-highest response is declining customer demand with 10 or 16.67%. It was discovered that the difficulties in creating new or innovative products cause the problem of the microenterprises’ operations. In adjusting to new methods of doing business, it is critical to be proactive, innovative, and optimistic.

**Table 9:** Determinants of the problems encountered by the respondents that lead to business failure in terms of the operational aspect

Category	f	%
Delay in delivering the supply material	8	13.33
Declining customers demand	10	16.67
Difficulties in making a new and innovative product	16	26.67
Obsolete (not updated) technology/machines	5	8.33
Distribution of products is hampered	8	13.33
The company has temporarily closed its doors due to new protocols and guidelines	13	21.67
Total	60	100.00

Table 10 presents the determinants of the problems encountered by handicraft business owners that led to business failure in terms of staffing and leadership, 15 or 25% are unable to adapt and adjust in reaction to a wide range of changes occurring around them, followed by 14 or 23.33% are owners’ inability to make a decision. The third highest response is business owners’ lack of vision and productivity with 12 or 20.00. It revealed that the cause of the problems encountered by the owners was their inability to adapt and adjust in reaction to a wide range of changes occurring around them. The SARS diseases had reduced the possibility of the microenterprises’ survivability in Africa continent; however by responding to market changes, micro-enterprises can still make a tremendous comeback [33]. Micro-enterprises must consequently possess entrepreneurial leadership traits to be successful. Tajudin et al. [24], identified the coping strategies of handicraft entrepreneurs despite the effect of COVID-19. Among the mentioned strategies was the ability to control stress, develop a strong spiritual relationship with God, apply problem-solving thinking skills, utilize social capital (offline and online), and optimize digital marketing [34].

**Table 10:** Determinants of the problems encountered by the respondents that lead to handicraft business failure in terms of staffing and leadership

Category	f	%
Large reduction in employees	5	8.33
Handicraft Business owners fail to pass information to their employees about policies and procedures	5	8.33
Handicraft Business owners lack vision and productivity	12	20.00
Used to bully and intimidate employees	2	3.33
Owners’ inability to make decision	14	23.33
Unable to adapt and adjust in reaction to a wide range of changes occurring around	15	25.00
Poor listening skills and lack of understanding	2	3.33
Quick to blame instead of taking responsibility	4	6.67
Others	1	1.67
Total	60	100.00

Table 11 shows the exit strategies employed by the micro-enterprises. The majority are bankrupt with 20 or 51.28%, followed by refinancing with 6 or 15.38%. The third-highest response is selling the business to family or friends with 5 or 12.82%. This revealed that most of the respondents filed for bankruptcy as a means of an exit strategy [5].

**Table 11:** Exit strategies employed by the respondents

Category	F	%
Bankruptcy	20	51.28
Initial Public Offering (IPO)	3	7.69
Hiring management	2	5.13
Refinancing	6	15.38
Liquidation	1	2.56
Sell the business to family or friend	5	12.82
Ownership is passed to the successor	2	5.13
Total	39	100.00

Sources: Table compiled by the author

## Conclusion and Recommendation

The researchers concluded the following:

Most of the problems encountered by the respondents in managing the enterprises during the pandemic in terms of the marketing aspect of the enterprise were limited to direct marketing, inefficient promotional activities, and lack of a marketing plan [9, 23]. The majority of respondents were sole proprietorships that had been in business for 2 to 5 years and had an initial capital of less than INR500,000.00. Considerably, in terms of financial aspects, owners’ revenue decreased, problems in budgeting and sourcing funds to grow the business, and large bills to pay were identified [24]. While in terms of operational aspects, starting with operating the business from home and using an online platform, difficulties in accessing workplaces, offices, factories, or warehouses, as well as operating for a shorter period. Problems encountered in terms of leadership and staffing were relocating employees to work from home, experiencing mental health problems because of the crisis, and fear of losing their job [32]. The majority of the respondent’ employed bankruptcy, refinancing, and selling the business to family or friends as a means of an exit strategy. Most of the determinants of problems that led to business failure during the pandemic were obstacles in the realignment of their respective goals owing to the pandemic, their ability to pay operational expenses, difficulties in product innovation, and their failure to react to the changes in the business environment. Exit strategies used by microenterprises included declaring bankruptcy, refinancing, and selling the business to family or friends [31].

Most of the determinants of the problems encountered by the microenterprises were challenges in re-aligning the goals due to the pandemic, the inability of business owners to pay ongoing expenses, difficulties in making a new and innovative product, and being unable to adapt and adjust to reaction to a wide range of changes occurring around them.

Based on the conclusions drawn, the following recommendations were proposed: handicraft enterprises should have a detailed process of converting parts of their operations into a flexible working setup. Considerably, small and medium entrepreneurs in the handicraft sector must consider the reinvention and innovation of their respective businesses, which could adapt to the changes in the business environment brought by the prevailing crisis; training in financial literacy for small entrepreneurs will further help them to budget and properly utilize financial assets which could anticipate difficulties in sustaining the operational expenses of the business; micro-entrepreneurs should also consider adopting updated technologies, especially in dissemination and utilization of information necessary for business operation; government agencies should have provisions for financial safety nets that will safeguard enterprises from the crises-led repercussions toward these enterprises [35, 36].

## Data Availability

The datasets used in this study are available upon request.

## Abbreviations

ADB: Asian development bank
MSME: Micro-small and medium enterprises
OECD: Organization for Economic Cooperation and Development
COVID: Coronavirus infectious disease
IEA: International energy agency
UNEP: United Nation Environmental Programme
EU: European Union
